# Plasma EVs Display Antigen-Presenting Characteristics in Patients With Allergic Rhinitis and Promote Differentiation of Th2 Cells

**DOI:** 10.3389/fimmu.2021.710372

**Published:** 2021-10-08

**Authors:** Shu-Bin Fang, Zhi-Rou Zhou, Ya-Qi Peng, Xiao-Qing Liu, Bi-Xin He, De-Hua Chen, Dong Chen, Qing-Ling Fu

**Affiliations:** Otorhinolaryngology Hospital, The First Affiliated Hospital, Sun Yat-sen University, Guangzhou, China

**Keywords:** allergic rhinitis, extracellular vesicles, Der p 1, antigen presentation, type 2 T helper cells

## Abstract

**Background:**

Allergic rhinitis (AR) is characterized by IgE-mediated mucosa response after exposure to allergens. Extracellular vesicles (EVs) are nano-size vesicles containing biological cargos for intercellular communications. However, the role of plasma EVs in pathogenesis of AR remains largely unknown.

**Methods:**

Plasma EVs from patients with AR were isolated, quantified, and characterized. The expression of Der p 1 and antigen-presenting molecules on EVs was determined by Western blot, flow cytometry, or ELISA. PKH26- and CFSE (carboxyfluorescein succinimidyl ester)-stained AR-EVs were used to determine the uptake of EVs by CD4^+^T cells and their effects on CD4^+^T cell proliferation, respectively.

**Results:**

Plasma EVs in healthy control (HC) and AR patients were similar in the concentration of particles, expression for specific EV markers, and both had structural lipid bilayer. However, the levels of Der p 1 on plasma EVs from both mild and moderate-severe AR patients were significantly higher than that on HC. The levels of antigen-presenting molecules on plasma EVs were similar from three subjects. Moreover, levels of Der p 1 on EVs in plasma, but not nasal secretion, were significantly associated with the symptom score of AR patients and level of plasma IL-13. Additionally, plasma EVs from patients with AR promoted the development of Th2 cells, while no effect was found on CD4^+^ T-cell proliferation.

**Conclusions:**

Plasma EVs derived from patients with AR exhibited antigen-presenting characteristics and promoted differentiation of Th2 cells, thus providing novel understanding of the pathogenesis of AR.

## Introduction

Allergic rhinitis (AR) is one of the most common allergic diseases that globally affects 10% to 40% of the population across all age groups (1). A number of evidences have shown that environmental allergens, such as house dust mites and pollen, are significant causes of the induction, persistence, and exacerbation of AR (2), leading to great impacts on patient’s quality of life and serious economic damages to our society (3). Although striking progresses have been made in the pathogenesis and treatment of AR during the past decades, morbidities of AR are still becoming increasingly higher and the clinical management for AR patients remained unsatisfied (4). Therefore, further studies are required to reveal the molecular and cellular mechanisms involved in the pathogenesis of AR, and thus providing potential therapeutic targets for treatment of AR.

Extracellular vesicles (EVs) are nano-vesicles that are substantially released by almost all cells and extensively involved in physiological and pathological processes in human bodies (5). Based on their size and mechanism of formation, these nano-sized particles are generally classified into exosomes released by multivesicular endosomes and microvesicles pinching off from plasma membrane (6). It has been widely demonstrated that EVs play crucial roles in local and systemic intercellular communication by transferring of their bioactive components, such as proteins, RNAs, and lipids (7). In the setting of allergic airway inflammation, EVs are secreted by various immunocytes such as antigen-presenting cells and airway epithelial cells, and presented in biofluids, including plasma and airway secretion (8). Previous studies have shown that miRNAs contained in EVs were able to exhibit immunoregulatory effects on allergic airway inflammation (9–11). Nevertheless, the roles of EVs in the interaction with immunocytes remain to be further elucidated.

In allergic airway inflammation, exposure to aeroallergen stimulates the activation and infiltration of CD4^+^ T cells, which are regarded as the key players in allergic responses (12). It has been well acknowledged that APCs, especially dendritic cells (DCs), are able to recognize and present foreign antigens to these CD4^+^ T cells for induction of allergen-specific Th2 responses. Besides, EVs released by APCs were considered as a novel and more complicated mode of interaction between APCs and T helper cells (5, 13). Particularly, it has been reported that allergen peptides, MHC II, and co-stimulatory molecules were present on EVs secreted by DCs and were able to mediate antigen-specific activation of T cells that were analogous to their parental APCs (14–17). In this scenario, EVs derived from APCs carrying antigen-presenting molecules are also present in airway lavage fluids (18, 19) and elicited antigen-specific immunoregulatory effects in allergic airway inflammation (19–21). In addition, it has been demonstrated that allergen peptides/MHC II-bearing EVs isolated from plasma of allergen-immunized mice were able to suppress delayed-type hypersensitivity inflammatory response in an antigen-specific manner (22). All these previous results suggest that EVs are able to play a significant role in antigen presentation of immune responses. However, it has never been reported whether or not allergen peptides/MHC II-bearing EVs are present in plasma of AR patients. Besides, the roles of plasma EVs on differentiation of Th2 cells remained to be further elucidated.

In this study, we sought to identify the presence of Der p 1 and antigen-presenting molecules on plasma EVs in AR patients, explore the relationship of EVs-carrying Der p 1 with the severity of AR patients, and reveal the effects of plasma EVs of AR patients on proliferation and differentiation of Th2 cells.

## Methods

### Subjects

Peripheral blood or nasal secretion was collected from patients with AR and healthy subjects for evaluation of level of EV-bearing Der p 1 in plasma or nasal secretion, isolation of plasma EVs, and *in vitro* cell culture experiments, as indicated in **Supplementary Table S1**. The detailed methods are presented in **Supplemental Materials**. Our study was approved by The Ethics Committee of The First Affiliated Hospital, Sun Yat-sen University. We obtained written informed consent from all the subjects before sample collection. Human blood buffy coats from “anonymous donors” were from Guangzhou Blood Center and the exemption of written informed consent was approved by The Ethics Committee of The First Affiliated Hospital, Sun Yat-sen University.

### Collection of Nasal Secretion

Nasal secretion of the subjects was collected as we previously reported (23). The detailed methods are presented in **Supplemental Materials**.

### Enzyme-Linked Immunosorbent Assay for Der p 1 on EVs

No commercial kits for determining the levels of Der p 1 on EVs are available. Therefore, we developed an ELISA assay for Der p 1 on EVs. The detailed methods are presented in **Supplemental Materials**. Level of IL-13 in plasma and cell culture supernatants was assayed using high-sensitivity commercial ELISA kit purchased from Neobioscience (Shenzhen, Guangdong, China) and Uncoated ELISA Kit purchased from Thermo Fisher Scientific (Waltham, MA, USA).

### Isolation of Plasma EVs

EVs were isolated from plasma of HC subjects (HC-EVs) and patients with moderate AR (M-AR-EVs) or moderate-severe AR (S-AR-EVs) by differential ultracentrifugation, as previously described with minor modifications (24). The detailed methods are presented in **Supplemental Materials**.

### Nanoparticle Tracking Analysis

Concentration and size distribution of plasma EVs were determined by nanoparticle tracking analysis using a NS300 Nanosight instrument (Malvern, UK). The detailed methods are presented in **Supplemental Materials**.

### Transmission Electron Microscopy

Morphologies of plasma EVs were observed using a TEM instrument (H7650; HITACHI, Tokyo, Japan) as we previously reported (25). The detailed methods are presented in **Supplemental Materials**.

### Western Blot Analysis

To determine levels of specific EV markers (CD9/CD63/CD81/Alix/TSG101) and molecules associated with antigen presentation (Der p 1/CD40/CD80/CD86/HLA-ABC/HLA-DR) in plasma EVs, Western blot analysis was conducted as we previously reported (25). The detailed methods are presented in **Supplemental Materials**.

### Staining of Plasma EVs With Carboxyfluorescein Succinimidyl Ester

The isolated plasma EVs were incubated with 20 nM CFSE for 1 h at room temperature, and the CFSE-stained plasma EVs were further isolated by ultracentrifugation. The CFSE-stained plasma EVs were significantly characterized by flow cytometry analysis (**Supplementary Figure S1**).

### Treatments of PBMCs or CD4^+^T Cells With EVs

Human PBMCs were isolated from buffy coats of anonymous donors provided by Guangzhou Blood Center by using density centrifugation with LSM™ Lymphocyte Separation Medium (MP Biomedicals, Santa Ana, CA, USA). Human CD4^+^ T cells were purified from PBMCs by using CD4^+^ T cell isolation kit (Miltenyi Biotec, Bergisch Gladbach, Germany) following manufacturer’s instructions.

The PBMCs or CD4^+^ T cells were seeded at a density of 5 × 10^5^ cells in 96-well plates and cultured with RPMI 1640 medium supplemented with 10% fetal bovine serum (FBS; Gibco, Carlsbad, CA, USA), 10 ng/ml IL-2 (Peprotech, Rocky Hill, NJ, USA) and penicillin/streptomycin (Gibco, Carlsbad, CA, USA) in the presence or absence of HC-EVs or AR-EVs at a concentration of 5 × 10^8^ particles/ml for 3 days. The cells were collected for Th1, Th2, and Th17 examination by flow cytometry analysis. The isolated CD4^+^ T cells were treated with CFSE-stained EVs and evaluated their uptake for EVs using flow cytometry or immunofluorescence.

For determination of the effects of plasma EVs on proliferation of CD4^+^ T cells, PBMCs were stained with CFSE, then stimulated with 5 mg/ml phytohemagglutinin (PHA), and treated with HC-EVs and AR-EVs as described above. The cells were then detected by flow cytometry analysis.

### Immunofluorescence

The detailed methods are presented in **Supplemental Materials**.

### Flow Cytometry Analysis

Levels of antigen-presenting molecules on plasma EVs CD4^+^ T cells treated with CFSE-stained EVs were analyzed as previously reported with minor modifications (26). The detailed methods are presented in **Supplemental Materials**.

### Statistical Analysis

Statistical analyses were performed with Prism software (GraphPad Software, La Jolla, CA, USA). All values were expressed as mean ± SEM for each group. Normal distribution was performed by using Kolmogorov-Smirnov test. For data with normal distribution, statistical significance was assessed by using one-way ANOVA with Tukey’s correction for multiple comparisons. For data with abnormal distribution, statistical significance was assessed by Mann-Whitney *U* test. Correlation analyses were performed using Pearson correlation coefficient. A *P* value less than 0.05 was considered statistically significant.

## Results

### The Characteristics of Plasma EVs Derived From Patients With AR

**Figure 1:**
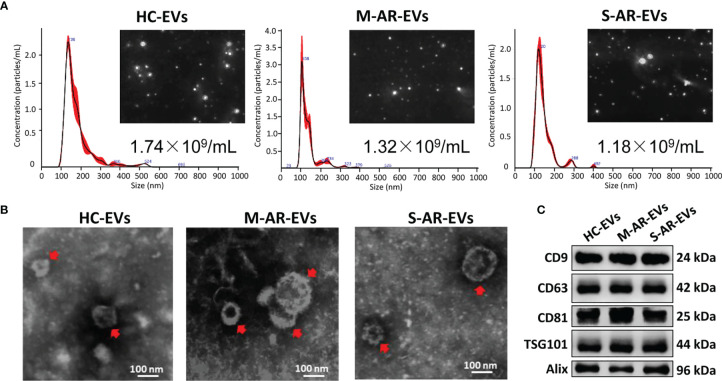
Characterization of plasma EVs. **(A)** Nanoparticle tracking analysis for determination of the particle numbers and size distribution of plasma EVs. **(B)** Transmission electron microscopy for characterization of the morphology of plasma EVs. **(C)** Plasma EVs isolated from HC subjects and patients with M-AR and S-AR were positive in the expression of specific EV markers. HC, Healthy control; M-AR, Mild allergic rhinitis; S-AR, Moderate-severe allergic rhinitis.

We first isolated plasma EVs from HC and AR patients that were sensitive to HDM by differential centrifugation, and we found no significant difference on the concentration of plasma EVs derived from HC, M-AR, and S-AR as determined by NTA (**Figure 1A**). All the three types of plasma EVs were nano-sized lipid-bilayer particles as determined by TEM (**Figure 1B**). Moreover, EVs isolated from HC and AR patients were both positive in specific EV markers, such as CD9, CD63, CD81, TSG101, and Alix, as determined by Western blot (**Figure 1C**). Our data suggested that EVs were successfully isolated from plasma of HC, M-AR, and S-AR subjects, which were similar in concentration, morphology, size, and expression of specific EV markers.

### Determination of Immunocyte-Related Markers on Plasma EVs

**Figure 2:**
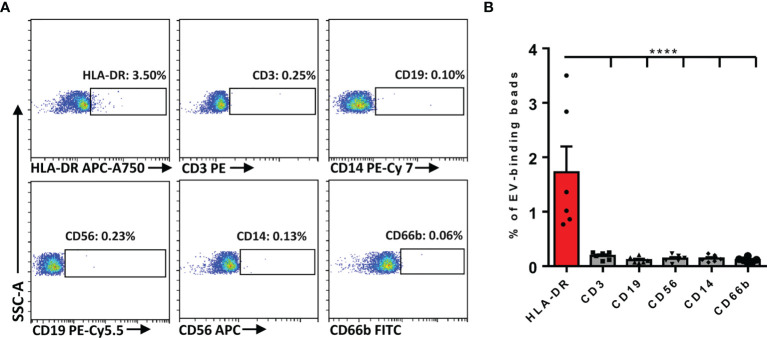
Determination of the immunocyte-associated markers on plasma EVs of AR patients. **(A)** Representative dot plots for flow cytometry analysis of immunocyte-related markers on plasma EVs. **(B)** Flow cytometry analysis of the expression of immunocyte-related markers on plasma EVs. Data are shown as mean ± SEM. ****p < 0.0001 by one-way ANOVA multiple comparison.

It has been well acknowledged that some cells were able to secrete EVs to carry corresponding specific markers of their parental cells into the circulation. Given that many immunocytes were involved in the inflammatory responses of AR, we first determined whether plasma EVs isolated from AR patients expressed some immunocyte-related markers. Flow cytometry analysis suggested that the markers about T cells (CD3^+^), B cells (CD19^+^), NK cells (CD56^+^), monocytes (CD14^+^), and granulocytes (CD66b^+^) were almost undetectable, while the expression of HLA-DR^+^ on plasma EVs were significantly detected (**Figures 2A, B**). Of note, APCs, especially DCs, are the key immunocytes that release HLA-DR^+^ EVs. Therefore, we speculated that circulating HLA-DR^+^ EVs could possibly be released by APCs in AR patients.

### Plasma EVs From AR Patients Expressed Antigen-Presenting Markers

**Figure 3:**
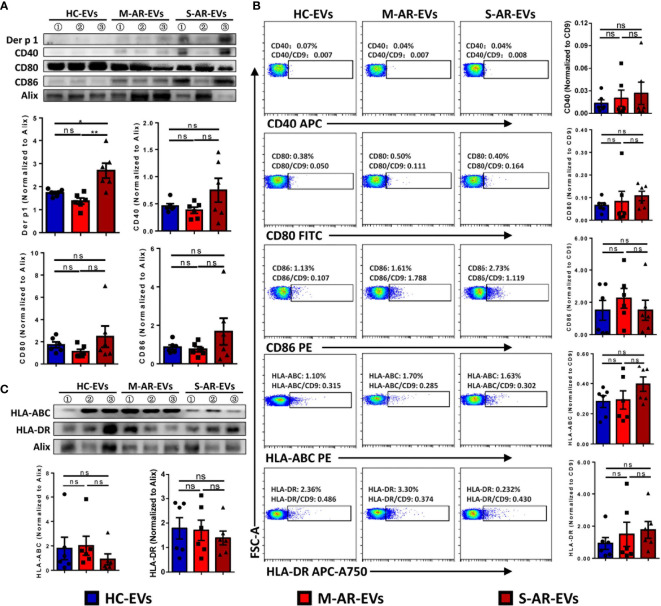
Determination of Der p 1, costimulatory molecules, and antigen presentation molecules on plasma EVs. The expression of costimulatory molecules **(A)** and antigen presentation molecules **(B)** was similar among plasma EVs derived from HC subjects and patients with M-AR and S-AR, while the expression of Der p 1 was significantly higher in patients with S-AR, as determined by Western blot. **(C)** No significant difference was found on levels of costimulatory and antigen presentation molecules on plasma EVs derived from HC subjects and patients with M-AR and S-AR, as suggested by flow cytometry analysis. EVs, Extracellular vesicles; HC, Healthy control; M-AR, Mild allergic rhinitis; ns, no significance; S-AR, Moderate-severe allergic rhinitis. Data are shown as mean ± SEM. Western blot of Der p 1, CD40, CD80, and CD86, as well as flow cytometry analysis of CD86 and HLA-ABC, were analyzed by Mann–Whitney *U* test; the other data were analyzed by one-way ANOVA multiple comparison. *p < 0.05. The data were combined from two independent experiments.

Then, we further investigated whether or not plasma EVs in AR patients elicited phenotypical signatures of APCs. Der p 1, one of the major allergens contained in HDM, can be presented by APCs to T cells and induces inflammatory responses. We thus first explored whether there was Der p 1 expression in AR-EVs. Our results showed that the level of Der p 1 on S-AR-EVs was significantly higher than that on HC-EVs and M-AR-EVs as detected by Western blot (**Figure 3A**). Furthermore, we found that costimulatory molecules, such as CD40, CD80 and CD86 (**Figure 3A**), as well as antigen presentation molecules, including HLA-ABC and HLA-DR (**Figure 3B**), were all presented in HC-EVs, M-AR-EVs, and S-AR-EVs by using Western blot but without significant difference among them (**Figures 3A, B**). In addition, we further confirmed that levels of costimulation and antigen presentation molecules on plasma EVs from HC and patients with M-AR or S-AR by bead-based flow cytometry and the results were almost similar to that of Western blot. Importantly, we also found that there was higher level of HLA-DR (1.77% ± 1.27%) than HLA-ABC (0.39% ± 0.12%) in plasma EVs of S-AR patients (**Figure 3C**), suggesting that plasma EVs may play more with CD4^+^ T cells. Because HLA-DR and HLA-ABC are mainly involved in the presentation of antigens to CD4^+^ and CD8^+^ T cells, respectively, our data at least partly suggested that plasma EVs of S-AR patients could play an important role in activation of T cells, especially CD4^+^ T cells. Collectively, our results have showed that plasma EVs derived from patients with AR especially severe patients with AR expressed Der p 1 and exhibited some APC properties. It suggests that plasma EVs derived from patients with AR might act as mini-APCs for antigen presentation in the pathogenesis of AR.

### The Level of Der p 1 on Plasma EVs Was Significantly Associated With the Severity of the Patients

**Figure 4:**
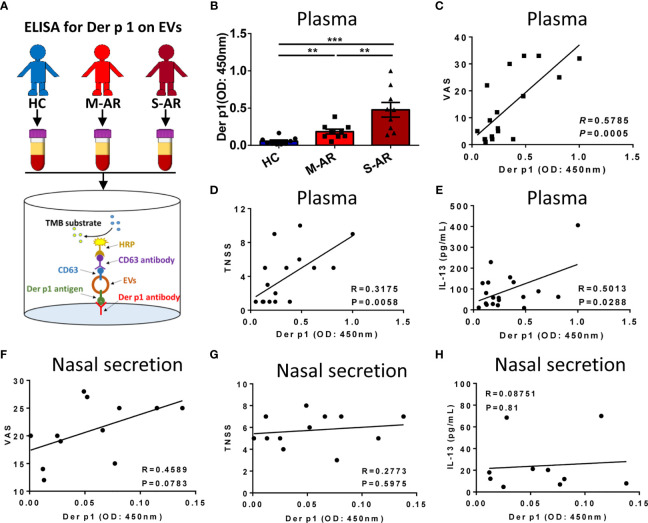
Detection of Der p 1-carrying EVs and the correlation between Der p 1^+^ EVs with the signs of the diseases and level of IL-13 in AR patients. **(A)** Schematic diagram of the ELISA for detection of Der p 1 on plasma EVs. **(B)** Levels of Der p 1 in patients with M-AR and S-AR were both significantly higher than HC subjects, as determined by ELISA. **(C–E)** Level of plasma EV-bearing Der p 1 in AR patients was significantly correlated with the VAS score **(C)**, TNSS score **(D)**, and level of IL-13 in plasma **(E)**. **(F–H)** Level of Der p 1 on nasal secretion-derived EVs in AR patients has no significant correlation with VAS score **(F)**, TNSS score **(G)**, and level of IL-13 in nasal secretion **(H)**. ELISA, Enzyme-linked immunosorbent assay; EVs, Extracellular vesicles; HC, Healthy control; M-AR, Mild allergic rhinitis; S-AR, Moderate-severe allergic rhinitis; TNSS, Total nasal symptom score; VAS, Visual analogue scale. **p < 0.01, ***p < 0.001 by one-way ANOVA multiple comparison. Correlation analyses were performed using Pearson correlation coefficient.

To investigate the association of Der p 1 on plasma EVs with the severity of AR patients, we next detected the level of EVs-associated Der p 1 in plasma of HC and AR patients by using an ELISA method designed by ourselves (**Figure 4A**). We found that the level of Der p 1 on plasma M-AR-EVs and S-AR-EVs was both significantly higher than that on HC-EVs, and higher level of Der p 1 was found on S-AR-EVs compared to M-AR-EVs (*p* < 0.01 or 0.001; **Figure 4B**). To understand whether level of Der p 1 on plasma EVs was related to the severity of AR patients, we further analyzed its correlation with the symptom scores. Our results showed that the level of Der p 1 on plasma EVs was significantly correlated with the visual analogue scale score (VAS; *p* = 0.0095, *R* = 0.5785; **Figure 4C**) and total nasal symptom score (TNSS; *p* = 0.0120, *R* = 0.3175; **Figure 4D**) of patients with AR. Besides, a significant correlation of plasma EV-carried Der p 1 and plasma IL-13 in AR patients was also observed (*p* = 0.0288, *R* = 0.5013; **Figure 4E**). Overall, our data demonstrated that Der p 1 on plasma EVs was highly associated with disease severity of AR. The above results mainly demonstrated that plasma EVs could be involved in the pathogenesis of AR.

We further evaluated the correlation of level of Der p 1 on EVs in nasal secretion with the symptom scores and type 2 cytokines in AR patients. We observed positive correlation between level of EV-carried Der p 1 in nasal secretion and VAS score but without significant difference (*p* = 0.07838, *R* = 0.4589; **Figure 4F**). Additionally, no significant correlation was found on the level of EV-carried Der p 1 and TNSS or IL-13 (**Figures 4G, H**). Our data suggested that there was no significant correlation between Der p 1 on EVs in nasal secretion with severity of AR patients.

### Plasma EVs From Patients With AR Promoted the Differentiation of Th2 Cells

**Figure 5:**
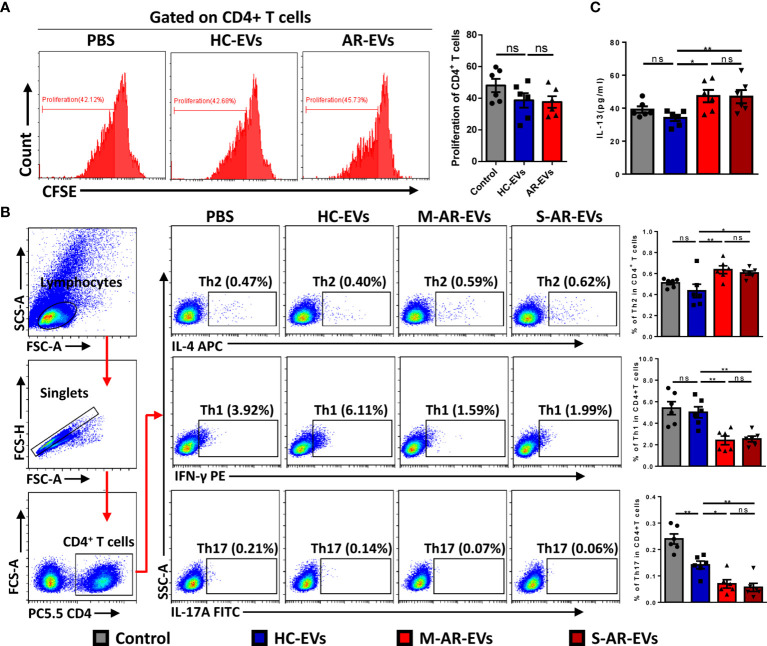
Effects of plasma EVs isolated from AR patients on the differentiation of T helper cells in PBMCs. **(A)** Flow cytometry analysis showed that no significant difference was observed on the proliferation of CD4^+^ T cells after treated with HC-EVs and AR-EVs. **(B)** Level of Th2 was increased in PBMCs treated with M-AR-EVs and S-AR-EVs compared with those treated with HC-EVs, while levels of Th1 and Th17 were oppositely reduced by M-AR-EVs and S-AR-EVs. **(C)** Level of IL-13 was significantly increased in the supernatants of AR-EV-stimulated PBMCs. EVs, Extracellular vesicles; HC, Healthy control; M-AR, Mild allergic rhinitis; S-AR, Moderate-severe allergic rhinitis; Th1, type 1 T helper cells; Th2, type 2 T helper cells; Th17, type 17 T helper cells; ns, no significance. Flow cytometry analyses of T helper cells and ELISA for IL-13 were analyzed by Mann–Whitney *U* test. Flow cytometry analyses of proliferation were analyzed by one-way ANOVA multiple comparison. *p < 0.05, **p < 0.01. The data were combined from two independent experiments.

To confirm the role of plasma EVs in pathogenesis of AR, we further determined the effect of AR-EVs on differentiation of T helper cells. The FMO for flow cytometry analysis of Th1, Th2 and Th17 cells was shown in supplementary **Figure S2**. We observed no significant effects on the proliferation of CD4^+^ T cells treated with both HC-EVs and AR-EVs (**Figure 5A**). Interestingly, both M-AR-EVs and S-AR-EVs were able to significantly increase the level of Th2 cells in PBMCs compared to HC-EVs, and oppositely decrease the levels of Th1 and Th17 cells, as shown by flow cytometry analysis (*p* < 0.05, **Figure 5B**). Consistently, we also found that level of IL-13 in the supernatants was significantly increased by the treatment of M-AR-EVs (*p* < 0.05) and S-AR-EVs compared to HC-EVs (*p* < 0.01, **Figure 5C**).

**Figure 6:**
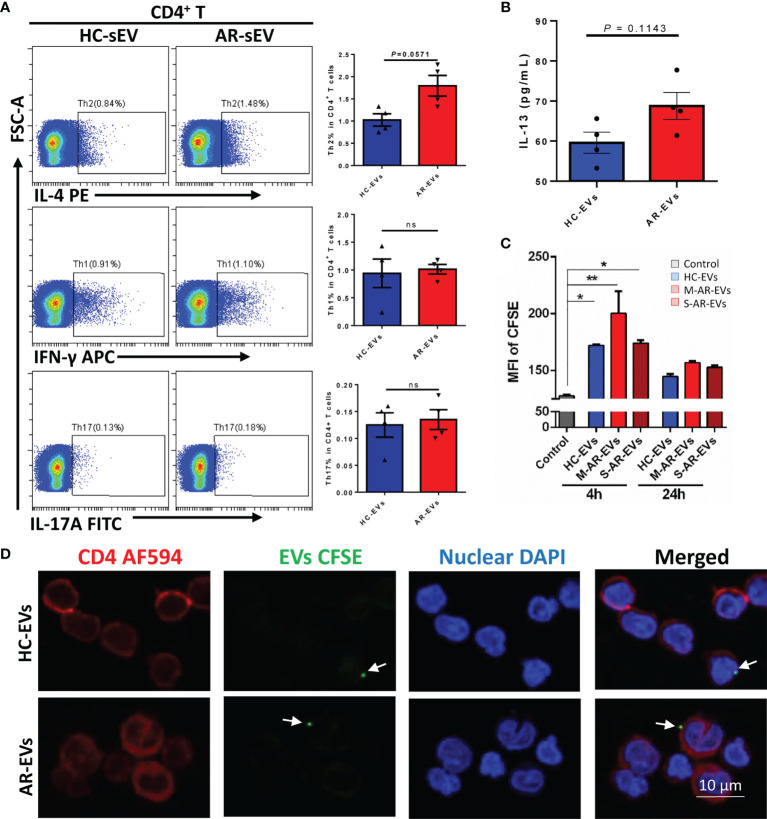
Plasma EVs isolated from AR patients promoted Th2 differentiation in isolated CD4^+^ T cells. **(A)** The levels of Th2, Th1 and Th17 with the treatment of AR-EVs or HC-EVs. **(B)** The level of IL-13 in the supernatants of AR-EV-stimulated CD4^+^ T cells. **(C)** Flow cytometry analysis of the uptake effects of CD4^+^ T cells on HC-EVs and AR-EVs. **(D)** Immunofluorescent images showing that both HC-EVs and AR-EVs were able to adhere to CD4^+^ T cells. EVs, extracellular vesicles; HC, healthy control, M-AR, Mild allergic rhinitis; S-AR, Moderate-severe allergic rhinitis; Th1, type 1 T helper cells; Th2, type 2 T helper cells; Th17, type 17 T helper cells; ns, no significance. Statistical analyses were performed by Mann–Whitney *U* test.

To confirm whether or not AR-EVs were able to regulate CD4^+^ T cells directly, we further incubated plasma EVs with isolated CD4^+^ T cells. We observed that administration of AR-EVs increased the levels of Th2 cells (*p* = 0.0571, **Figure 6A**) and IL-13 (*p* = 0.1143, **Figure 6B**) compared to the treatment of HC-EVs but without significant differences. No significant decrease was observed on levels of Th1 and Th17 cells in AR-EVs stimulated purified CD4^+^ T cells (**Figure 6A**).

Using CFSE-labeled EVs, we found that level of MFI of CFSE in CD4^+^ T cells were significantly increased at 4 h but not at 24 h after the treatments of HC-EVs and AR-EVs especially for M-AR-EVs (*p* < 0.05 or 0.01, **Figure 6C**), suggesting the uptake of EVs by CD4^+^ T cells. Moreover, we found that there were immunofluorescent EVs surrounding or binding to CD4^+^ T cells both for HC-EVs and AR-EVs (**Figure 6D**). It suggests that AR-EVs could possibly interact with or taken up by CD4^+^ T cells to promote Th2 differentiation. Our data demonstrated that plasma EVs could be a critical factor in promoting the differentiation of Th2 cells.

## Discussion

In our study, for the first time, we revealed that Der p 1 was presented on plasma EVs of patients with AR, and that phenotypical APC signatures were found on AR plasma EVs. Moreover, the level of Der p 1 on EVs in plasma was highly associated with the symptom score and type 2 cytokines in AR patients. Importantly, we demonstrated that plasma EVs from AR patients was able to promote the development of Th2 cells.

It has been widely reported that EVs were presented in body fluids such as plasma and airway secretion, and these EVs were intensively involved in the pathogenesis of different diseases (27). In allergic diseases, previous studies have demonstrated the differential expression of miRNAs in EVs isolated from airway secretion and plasma, which could play a significant role in the immunoregulation on their recipient cells (9, 11, 28). Besides miRNAs, EVs also contain many other bioactive components such as proteins. However, to our knowledge, the role of proteins contained in plasma EVs has never been reported in allergic airway diseases. In our studies, we set out to isolate EVs from plasma of AR patients, and preliminarily determined levels of typical markers of different immunocytes on the isolated plasma EVs to investigate the possible cellular sources of plasma EVs. Interestingly, we found that HLA-DR, one of the representative markers of APCs, can be detected in plasma EVs of AR patients. These results led us to the hypothesis that APCs may release EVs into circulation of AR patients and mediated allergic responses. To confirm our hypothesis, we further investigated the expression of Der p 1 and antigen presentation-associated molecules on plasma EVs of HDM-sensitive AR patients. As expected, we observed that Der p 1 on S-AR-EVs was significantly higher than that on M-AR-EVs and HC-EVs, and expression of costimulatory and antigen presentation molecules was also detected. However, no significant difference was found on levels of costimulatory and antigen presentation molecules in means of Western blot or flow cytometry analysis. This in part could be because that release of EVs from APCs by allergen stimulation was not significant enough to be identified by the detection methods we applied. Our findings suggested that circulating EVs in AR patients would possibly work as mini-APCs in antigen-specific immune responses.

Antigen presentation has been considered as an important aspect of the pathophysiological mechanism of AR, in which APCs acted as the major sentinel in the immune responses. Previous studies have shown that APCs, such as dendritic cells and B cells, were able to release “mini-APC-like” EVs and mediated antigen-specific activation of CD4^+^ T cells *in vitro* (13–15). Some studies also reported that systemic administration of EVs isolated from APCs were able to interact with cells far away from the injection site (29, 30). However, it remains largely unknown whether EVs secreted by APCs in airway were able to be introduced into the circulation for immune responses. In our study, we found that level of Der p 1 on plasma EVs in AR patients was highly correlated with symptom severity and type 2 cytokines in plasma. Our findings suggested that EVs, unlike APCs that require specific chemokines and their receptors for migration, could in part pass through impermeable blood vessels and enter the circulation for mediation of antigen-specific responses. As far as we know, our study was the first time to reveal the presentation of foreign antigen peptide and APC characteristics on plasma EVs in AR patients, which provides novel understanding for the pattern of antigen presentation in allergic responses. Besides, we found that Der p 1 was presented on EVs in secretion of nasal cavity, the local site of immune responses in AR patients. However, we found no significant correlation of EV-carrying Der p 1 with the symptom severity and type 2 cytokine in AR patients. This might be because the concentration of EVs in nasal secretion was greatly influenced by the amount of nasal secretion. Since we could not obtain enough nasal secretion for isolation of EVs, we did not go further to investigate whether EVs in nasal secretion exhibited APC characteristics as plasma EVs did in AR patients. Overall, our findings suggested that Der p 1 in EVs derived from nasal secretion showed less importance than plasma EVs in AR patients.

It has been well established that levels of Th2 cells were significantly increased in circulation of AR patients and were significantly correlated with the symptom severity of AR patients (31). The differentiation of T helper cells was typically considered to be mediated by soluble cytokines produced by immunocytes like APCs in non-lymphoid tissues (32). In our study, we demonstrated that the plasma EVs of AR patients were able to promote the activation of Th2 cells as demonstrated by flow cytometry analysis of Th cells and ELISA for IL-13. It may be because that circulating EVs in AR patients possessed APC signatures. However, circulating EVs showed no effects on the proliferation of CD4^+^ T cells. Since AR is mainly Th2-dominant, we did not measure the levels of the Th1- or Th17-associated cytokines in plasma of AR patients. In our study, we found that AR-EVs showed effects on Th2 differentiation but not on proliferation of CD4^+^ T cells. However, it still cannot be excluded that AR-EVs might elicit effects on CD4^+^ T-cell proliferation under the treatments of higher concentration of AR-EVs. Also, our data revealed the possibility that plasma EVs could be a novel factor for Th2 differentiation in the circulation during the pathogenesis of AR. It has been established that the effects of Der p 1-bearing EVs released by APCs were able to directly interact with CD4^+^ T cells or indirectly be taken up and presented by APCs for promoting the differentiation of Th2 cells (13). Our results have showed that AR-EVs promote the differentiation of Th2 cells and increase level of IL-13 in PBMCs. Similarly, AR-EVs also increased the levels of Th2 cells and IL-13 in purified CD4^+^ T cells, though no significant effects were observed due to the limited sample size. We further confirmed that AR-EVs were able to adhere to or be taken up by CD4^+^ T cells, which at least in part demonstrated that AR-EVs were possibly able to interact directly with CD4^+^ T cells for promoting differentiation of Th2 cells.

We acknowledge that there are some limitations in our study. Firstly, the sample size of our study is relatively small due to limited collection of human blood samples. Besides, we only showed that plasma EVs of AR patients were able to promote the differentiation of Th2 cells. However, whether or not the effects of Der p 1-carrying plasma EVs on activation of Th2 cells were antigen-dependent remains to be further elucidated. Also, we did not investigate the presence of other antigen peptides on plasma EVs of AR patients, because most of the AR patients in Southern China were sensitive to HDM and we were not able to recruit enough AR patients that were sensitive to other allergens.

## Conclusions

In conclusion, we found that plasma EVs in AR patients carried antigen peptides and exhibited antigen-presenting characteristics, level of Der p 1 on plasma EVs was significantly correlated with symptom severity and type 2 inflammatory cytokines, and AR-EVs were able to activate Th2 cells. Our study provided novel understanding of the pattern of antigen presentation in allergic responses of AR patients, which could work as novel therapeutic targets for AR patients in the future.

## Data Availability Statement

The raw data supporting the conclusions of this article will be made available by the authors, without undue reservation.

## Ethics Statement

The studies involving human participants were reviewed and approved by the Ethics Committee of The First Affiliated Hospital, Sun Yat-sen University. The patients/participants provided their written informed consent to participate in this study.

## Author Contributions

S-BF and Z-RZ helped in collection and/or assembly of data and manuscript writing. Y-QP helped in the collection of data. X-QL and B-XH prepared the sEV and performed the experiments using Nanosight. D-HC and DC helped in the collection of samples from the patients. Q-LF helped in concept and design, data analysis, manuscript writing, and final approval of the manuscript. All authors contributed to the article and approved the submitted version.

## Funding

This study was supported by grants from the National Natural Science Foundation, China (grant nos. 81770984, 81970863, and 8210040172), Guangdong Basic and Applied Basic Research Foundation (grant no. 2020A1515110794), and China Postdoctoral Science Foundation (grant no. 2021M693639).

## Conflict of Interest

The authors declare that the research was conducted in the absence of any commercial or financial relationships that could be construed as a potential conflict of interest.

## Publisher’s Note

All claims expressed in this article are solely those of the authors and do not necessarily represent those of their affiliated organizations, or those of the publisher, the editors and the reviewers. Any product that may be evaluated in this article, or claim that may be made by its manufacturer, is not guaranteed or endorsed by the publisher.

## References

[1] Bousquet J Khaltaev N Cruz AA Denburg J Fokkens WJ Togias A. Allergic Rhinitis and its Impact on Asthma (ARIA) 2008 Update (in Collaboration With the World Health Organization, GA(2)LEN and AllerGen). Allergy (2008) 63(Suppl 86):8–160. doi: 10.1111/j.1398-9995.2007.01620.x 18331513

[2] Gauvreau GM El-Gammal AI Byrne PM. Allergen-Induced Airway Responses. Eur Respir J (2015) 46(3):819. doi: 10.1183/13993003.00536-2015 26206871

[3] Meltzer EO. Allergic Rhinitis: Burden of Illness, Quality of Life, Comorbidities, and Control. Immunol Allergy Clin North Am (2016) 36(2):235–48. doi: 10.1016/j.iac.2015.12.002 27083099

[4] Zhang Y Zhang L. Increasing Prevalence of Allergic Rhinitis in China. Allergy Asthma Immunol Res (2019) 11(2):156–69. doi: 10.4168/aair.2019.11.2.156 PMC6340797 30661309

[5] Robbins PD Morelli AE. Regulation of Immune Responses by Extracellular Vesicles. Nat Rev Immunol (2014) 14(3):195–208. doi: 10.1038/nri3622 24566916 PMC4350779

[6] Thery C Witwer KW Aikawa E Alcaraz MJ Anderson JD Andriantsitohaina R. Minimal Information for Studies of Extracellular Vesicles 2018 (MISEV2018): A Position Statement of the International Society for Extracellular Vesicles and Update of the MISEV2014 Guidelines. J Extracell Vesicles (2018) 7(1):1535750. doi: 10.1080/20013078.2018.1535750 30637094 PMC6322352

[7] Tkach M Thery C. Communication by Extracellular Vesicles: Where We Are and Where We Need to Go. Cell (2016) 164(6):1226–32. doi: 10.1016/j.cell.2016.01.043 26967288

[8] Sangaphunchai P Todd I Fairclough LC. Extracellular Vesicles and Asthma: A Review of the Literature. Clin Exp Allergy (2020) 50(3):291–307. doi: 10.1111/cea.13562 31925972

[9] Levanen B Bhakta NR Torregrosa Paredes P Barbeau R Hiltbrunner S Pollack JL. Altered microRNA Profiles in Bronchoalveolar Lavage Fluid Exosomes in Asthmatic Patients. J Allergy Clin Immunol (2013) 131(3):894–903. doi: 10.1016/j.jaci.2012.11.039 23333113 PMC4013392

[10] Simpson LJ Patel S Bhakta NR Choy DF Brightbill HD Ren X. A microRNA Upregulated in Asthma Airway T Cells Promotes TH2 Cytokine Production. Nat Immunol (2014) 15(12):1162–70. doi: 10.1038/ni.3026 PMC4233009 25362490

[11] Sanchez-Vidaurre S Eldh M Larssen P Daham K Martinez-Bravo MJ Dahlen SE. RNA-Containing Exosomes in Induced Sputum of Asthmatic Patients. J Allergy Clin Immunol (2017) 140(5):1459–61.e2. doi: 10.1016/j.jaci.2017.05.035 28629752

[12] Ling MF Luster AD. Allergen-Specific CD4(+) T Cells in Human Asthma. Ann Am Thorac Soc (2016) 13 Suppl 1(Suppl 1):S25–30. doi: 10.1513/AnnalsATS.201507-431MG PMC5015731 27027948

[13] Lindenbergh MFS Stoorvogel W. Antigen Presentation by Extracellular Vesicles From Professional Antigen-Presenting Cells. Annu Rev Immunol (2018) 36:435–59. doi: 10.1146/annurev-immunol-041015-055700 29400984

[14] Admyre C Bohle B Johansson SM Focke-Tejkl M Valenta R Scheynius A. B Cell-Derived Exosomes can Present Allergen Peptides and Activate Allergen-Specific T Cells to Proliferate and Produce TH2-Like Cytokines. J Allergy Clin Immunol (2007) 120(6):1418–24. doi: 10.1016/j.jaci.2007.06.040 17868797

[15] Luketic L Delanghe J Sobol PT Yang P Frotten E Mossman KL. Antigen Presentation by Exosomes Released From Peptide-Pulsed Dendritic Cells Is Not Suppressed by the Presence of Active CTL. J Immunol (2007) 179(8):5024–32. doi: 10.4049/jimmunol.179.8.5024 17911587

[16] Raposo G Nijman HW Stoorvogel W Liejendekker R Harding CV Melief CJ. B Lymphocytes Secrete Antigen-Presenting Vesicles. J Exp Med (1996) 183(3):1161–72. doi: 10.1084/jem.183.3.1161 PMC2192324 8642258

[17] Qiu S Du Y Duan X Geng X Xie J Gao H. Cytotoxic T Lymphocytes Mediate Chronic Inflammation of the Nasal Mucosa of Patients With Atypical Allergic Rhinitis. N Am J Med Sci (2011) 3(8):378–83. doi: 10.4297/najms.2011.3378 PMC3234139 22171246

[18] Admyre C Grunewald J Thyberg J Gripenback S Tornling G Eklund A. Exosomes With Major Histocompatibility Complex Class II and Co-Stimulatory Molecules Are Present in Human BAL Fluid. Eur Respir J (2003) 22(4):578–83. doi: 10.1183/09031936.03.00041703 14582906

[19] Prado N Marazuela EG Segura E Fernández-García H Villalba M Théry C. Exosomes From Bronchoalveolar Fluid of Tolerized Mice Prevent Allergic Reaction. J Immunol (2008) 181: (2):1519–25. doi: 10.4049/jimmunol.181.2.1519 18606707

[20] Shin TS Kim JH Kim YS Jeon SG Zhu Z Gho YS. Extracellular Vesicles are Key Intercellular Mediators in the Development of Immune Dysfunction to Allergens in the Airways. Allergy (2010) 65(10):1256–65. doi: 10.1111/j.1398-9995.2010.02359.x PMC3066408 20337607

[21] Torregrosa Paredes P Esser J Admyre C Nord M Rahman QK Lukic A. Bronchoalveolar Lavage Fluid Exosomes Contribute to Cytokine and Leukotriene Production in Allergic Asthma. Allergy (2012) 67(7):911–9. doi: 10.1111/j.1398-9995.2012.02835.x 22620679

[22] Kim SH Bianco NR Shufesky WJ Morelli AE Robbins PD. MHC Class II+ Exosomes in Plasma Suppress Inflammation in an Antigen-Specific and Fas Ligand/Fas-Dependent Manner. J Immunol (2007) 179(4):2235–41. doi: 10.4049/jimmunol.179.4.2235 17675484

[23] Peng YQ Qin ZL Fang SB Xu ZB Zhang HY Chen D. Effects of Myeloid and Plasmacytoid Dendritic Cells on ILC2s in Patients With Allergic Rhinitis. J Allergy Clin Immunol (2019) 145(3):855–67. doi: 10.1016/j.jaci.2019.11.029 31812574

[24] Thery C Amigorena S Raposo G Clayton A. Isolation and Characterization of Exosomes From Cell Culture Supernatants and Biological Fluids. Curr Protoc Cell Biol (2006) 3.22.1–29. doi: 10.1002/0471143030.cb0322s30. 18228490

[25] Fang S-B Zhang H-Y Wang C He B-X Liu X-Q Meng X-C. Small Extracellular Vesicles Derived From Human Mesenchymal Stromal Cells Prevent Group 2 Innate Lymphoid Cell-Dominant Allergic Airway Inflammation Through Delivery of miR-146a-5p. J Extracell Vesicles (2020) 9(1):1723260. doi: 10.1080/20013078.2020.1723260 32128074 PMC7034457

[26] Zhang F Li R Yang Y Shi C Shen Y Lu C. Specific Decrease in B-Cell-Derived Extracellular Vesicles Enhances Post-Chemotherapeutic CD8(+) T Cell Responses. Immunity (2019) 50(3):738–50.e7. doi: 10.1016/j.immuni.2019.01.010 30770248

[27] Boukouris S Mathivanan S. Exosomes in Bodily Fluids are a Highly Stable Resource of Disease Biomarkers. Proteomics - Clin Applications (2015) 9(3-4):358–67. doi: 10.1002/prca.201400114 PMC5502131 25684126

[28] Kimura K Hohjoh H Fukuoka M Sato W Oki S Tomi C. Circulating Exosomes Suppress the Induction of Regulatory T Cells via Let-7i in Multiple Sclerosis. Nat Commun (2018) 9(1):17. doi: 10.1038/s41467-017-02406-2 29295981 PMC5750223

[29] Morelli AE Larregina AT Shufesky WJ Sullivan MLG Stolz DB Papworth GD. Endocytosis, Intracellular Sorting, and Processing of Exosomes by Dendritic Cells. Blood (2004) 104(10):3257–66. doi: 10.1182/blood-2004-03-0824 15284116

[30] Saunderson SC Dunn AC Crocker PR McLellan AD. CD169 Mediates the Capture of Exosomes in Spleen and Lymph Node. Blood (2014) 123(2):208–16. doi: 10.1182/blood-2013-03-489732 PMC3888287 24255917

[31] Horiguchi S Okamoto Y. Role of T Cells in Allergic Rhinitis. Clin Exp Allergy Rev (2005) 5(2):64–7. doi: 10.1111/j.1365-2222.2005.0087.x

[32] Saravia J Chapman NM Chi H. Helper T Cell Differentiation. Cell Mol Immunol (2019) 16(7):634–43. doi: 10.1038/s41423-019-0220-6 PMC6804569 30867582

