# Supplementary material for: Plasma EVs Display Antigen-Presenting Characteristics in Patients With Allergic Rhinitis and Promote Differentiation of Th2 Cells
Source: Front Immunol. 2021 Oct 8;12:710372. doi: 10.3389/fimmu.2021.710372 (PMC8531542; doi:10.3389/fimmu.2021.710372)
Supplement: Supplementary file 1 [file DataSheet_1.docx]

Supplementary Material

**Plasma EVs display antigen presenting characteristics in patients with allergic rhinitis and promote differentiation of Th2 cells**

**Shu-Bin Fang ^1^**^‡^**, Zhi-Rou Zhou ^1^**^‡^**, Ya-Qi Peng ^1^, Xiao-Qing Liu ^1^, Bi-Xin He ^1^, De-Hua Chen ^1^, Dong Chen ^1^, Qing-Ling Fu ^1*^**

^1^ Otorhinolaryngology Hospital, The First Affiliated Hospital, Sun Yat-sen University, 58 Zhongshan Road II, Guangzhou, China;

‡ These authors contributed equally to this work as first authors.

*** Correspondence:**Prof. Qing-Ling Fu
[fuqingl@mail.sysu.edu.cn](mailto:fuqingl@mail.sysu.edu.cn)

# Supplementary Methods

**Subjects**

All the patients with allergic rhinitis (AR) were recruited according to the diagnostic criteria Initiative on Allergic Rhinitis and its Impact on Asthma (ARIA) 2010 (1), including the history of nasal symptoms (rhinorrhea, nasal itching, nasal obstruction, sneezing and postnasal drip) and positive for Dermatophagoides pteronyssinus (Der p 1) as determined by skin prick test (SPT) to house dust mites (HDM) and specific IgE tests (>0.35 IU/mL, Pharmacia CAP System; Pharmacia Diagnostics, Uppsala, Sweden). In some experiments, AR patients were further divided into mild AR (M-AR) and moderate-severe AR (S-AR) based on the severity of symptoms as defined in ARIA 2010. Briefly, those patients without any of the symptoms, including sleep disturbance, impairment of daily activities, leisure and/or sport, Impairment of school or work or troublesome symptoms, were considered as M-AR, and those with any of the above symptoms were considered as S-AR. The healthy control subjects did not report any nasal symptoms of AR or have a history of AR, and were negative for SPT to HDM and serum specific IgE to any of the allergens tested. All the participants did not take antihistamines, tropical or systemic steroids and biologics for at least 1 month, and had no histories of any other diseases or smoking. No differences in occupational exposure to aeroallergens or residential areas between HC, M-AR and S-AR groups

**Collection of nasal secretion**

Nasal secretion of the subjects was collected as we previously reported (2). Briefly, a small saline paper disk was placed on the anterior end of left inferior turbinate for 5 min. Then, transferred the paper disk into a syringe barrel, squeezed the paper disk with a syringe piston, and collected the nasal secretion using a 1.5 mL tube. The samples were centrifuged at 300 g to remove cells and stored at -80 ℃ for further analyses.

**Enzyme-linked immunosorbent assay (ELISA) for Der p 1 on EVs**

No commercial kits about the levels of Der p 1 on EVs are available. Therefore, we developed an ELISA assay for Der p 1 on EVs. For detection of Der p 1 on EVs in plasma, nasal secretion or cell culture supernatant, MaxiSorp ELISA plates (Thermo Fisher Scientific, Carlsbad, CA, USA) were coated with 0.1 μg/well rabbit anti-Der p 1 (RayBiotech, Peachtree Corners, GA, USA) that were diluted with 100 μL 0.2 M sodium bicarbonate, and incubated at 4 ℃ overnight. The free binding sites were further blocked with 100 μL 5% bovine serum albumin (BSA) at room temperature for 1 h. Then, 50 μL of plasma or nasal secretion samples were loaded to each well and incubated for 2 h at room temperature. After 5 washes, 100 μL of biotin anti-human CD63 antibody (Biolegend, San Diego, CA, USA), diluted at 1:100 with PBS containing 1% BSA, were added to each well and incubated for 1 h at room temperature. Afterwards, 100 μL of horseradish peroxidase-conjugated streptavidin (R&D Systems, Minneapolis, MN, USA), diluted with PBS containing 1% BSA at a ratio of 1:100, were added to each well and incubated for 30 min at room temperature. Finally, plates were developed with Substrate Solution (R&D Systems, Minneapolis, MN, USA) for 5 min, stopped with 2 N H_2_SO_4_ (R&D Systems, Minneapolis, MN, USA) and detected on a multi-mode microplate reader (Synergy HT; Biotech, Winooski, VT, USA).

**Isolation of plasma EVs**

EVs were isolated from plasma of HC subjects (HC-EVs) and patients with M-AR (M-AR-EVs) or S-AR (S-AR-EVs) by differential ultracentrifugation, as previously described with minor modifications (3). Briefly, cells were removed from blood samples by centrifugation at 600 g for 10 min. The plasma was collected, diluted with ice-cold PBS at 1:5, and further centrifuged at 2000 g for 20 min to remove cellular debris. Then the supernatants were centrifuged at 12000 g for 30 min to deplete large vesicles, followed by sequential ultracentrifugation at 110000 g for 2 h and 70 min, respectively, to pellet EVs. All the procedures were performed by using aseptic techniques and all the centrifugation was conducted at 4 ℃. The EV samples underwent Nanoparticle Tracking Analysis for detection of particle numbers before being stored at -80 ℃.

**Nanoparticle tracking analysis (NTA)**

Concentration and size distribution of plasma EVs were determined by nanoparticle tracking analysis using a NS300 Nanosight instrument (Malvern, UK). Briefly, EV samples were diluted with particle-free PBS at the appropriate proportion to meet the recommended range (1-10×10^8^ particles/mL), and then the diluted samples were loaded into the sample chamber using a 1 mL syringe. Then three video recordings were captured for 60 s each, which were analyzed using NTA 2.3 build 17 software (Malvern, UK). The post-acquisition settings were optimized and stayed the same for the analysis of each sample.

**Transmission electron microscopy (TEM)**

Morphologies of plasma EVs were observed using a TEM instrument (H7650; HITACHI, Tokyo, Japan) as we previously reported (4). Briefly, 10 µL of EV samples were fixed with 2% glutaraldehyde (Sigma, Saint Louis, MO, USA) and deposited onto carbon-coated electron microscopy grids. The grids were then washed three times with ddH_2_O, followed by incubation with 3% uranyl acetate (Sigma, Saint Louis, MO, USA, pH = 7.0) for 5 min and another three washes with ddH_2_O. Finally, the grids were observed and photographed technically supported by Guangdong Institute of Microbiology.

**Western blot analysis**

To determine levels of specific EV markers (CD9/CD63/CD81/Alix/TSG101) and molecules associated with antigen presentation (Der p 1/CD40/CD80/CD86/HLA-ABC/HLA-DR) in plasma EVs, western blot analysis were conducted as we previously reported (4). Briefly, 20 µg of EV proteins were separated using 12% SDS-PAGE and transferred onto polyvinylidene difluoride membranes (Roche Diagnostics, Mannheim, Germany). The membranes were then blocked with 5% skim milk for 1 h at room temperature, and incubated correspondingly with the primary rabbit antibodies overnight at 4 ℃, followed by incubation with HRP conjugated anti-rabbit IgG at room temperature for 1 h. The blots were finally developed with Enhanced Chemiluminescence Plus (Millipore Corporation, Billerica, MA, USA) using ChemiDoc™ Touch Imaging System (Bio-Rad, Hercules, CA, USA). The primary antibody to Der p 1 was purchased in RayBiotech, Peachtree Corners, GA, USA, and the other primary antibodies were all purchased in Abcam, Cambridge, UK.

**Immunofluorescence**

Both HC-EVs and AR-EVs were stained with Carboxyfluorescein Succinimidyl Ester (CFSE, Sigma, Saint Louis, MO, USA) following manufacturer’s instruction, and incubated with CD4^+^ T cells. The cells were washed with PBS, fixed with 4% paraformaldehyde for 20 min, and smeared onto slides. For evaluation of the interaction of plasma EVs and CD4^+^ T cells, cells were stained with rabbit antibodies to CD4 (Abcam, Cambridge, UK), followed by staining of AF594-conjugated Goat anti-Rabbit IgG (H+L) Secondary Antibody (Invitrogen, Eugene, OR, USA).

**Flow cytometry analysis**

Levels of antigen presenting molecules on plasma EVs were analyzed as previously reported with minor modifications (5). Briefly, EVs were coated onto 4-mm-diameter aldehyde-sulfate latex beads (Invitrogen, New York, NY, USA), blocked with 100 mM glycine for 30 min, and stained with fluorescence-labeled antibodies to CD40, CD80, CD86, HLA-ABC and HLA-DR correspondingly. T helper cells were analyzed as we previously reported (6). Briefly, cells were stimulated with 50 ng/mL phorbol myristate acetate (Sigma, St. Louis, MO, USA), 1000 ng/mL ionomycin (St. Louis, Sigma, MO, USA) and Monensin (1:1000; Thermo Fisher Scientific, Carlsbad, CA, USA) for 5 h, followed by cell surface staining of anti-CD4 and intracellular staining of anti-IFN-γ, anti-IL-4 and anti-IL-17A. CD4^+^ T cells treated with CFSE-stained EVs were collected and stained with anti-CD4 antibody. All the cells were analyzed using a CytoFLEX Flow Cytometer (Beckman Coulter, Hercules, CA, USA), and gated according to the FMO (Supplementary Fig.2). All the antibodies were purchased from Thermo Fisher Scientific, San Diego, CA, USA.

# Supplementary Tables

**Supplementary Table 1** Baseline characteristics of the participants’ demographics, total IgE, specific IgE and symptom score involved in this study.

| **Characteristic** | **HC subjects** | **AR patients** | | ***P* value** |
| --- | --- | --- | --- | --- |
|  |  | **Mild** | **Moderate-Severe** |  |
| No. of patients | 9 | 9 | 9 | - |
| Age (y) | 26.33±0.60 | 24.56±0.81 | 24.78±1.42 | ns |
| Sex: female/male | 2/7 | 3/6 | 3/6 | ns |
| SPT result, (positive/subjects tested) | 0 (0%) | 9 (100%) | 9 (100%) | - |
| VAS score | 0 | 3.11±0.59 | 23.78±3.05 | ＜0.0001 |
| TNSS score | 0 | 1.11±0.11 | 6.33±0.80 | ＜0.0001 |

Abbreviations: *AR* allergic rhinitis, *sIgE* specific IgE, *SPT* skin prick test, *tIgE* total IgE, *TNSS* total nasal symptom score, *VAS* visual analogue scale score.


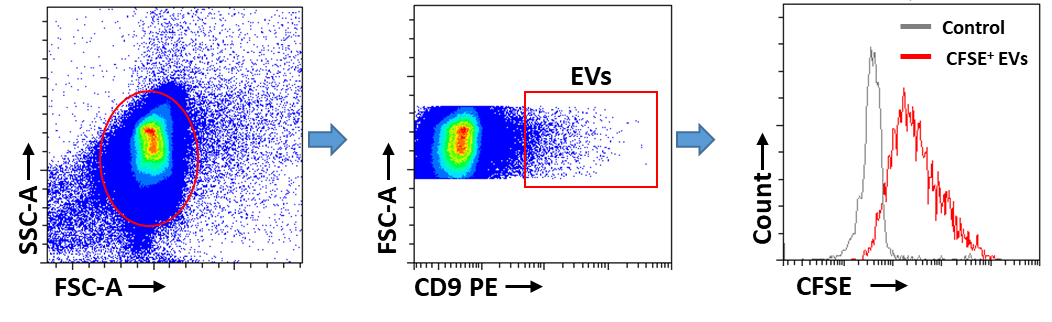


**Supplementary Fig.1 Characterization of CFSE-labelled plasma EVs by flow cytometry analysis.**


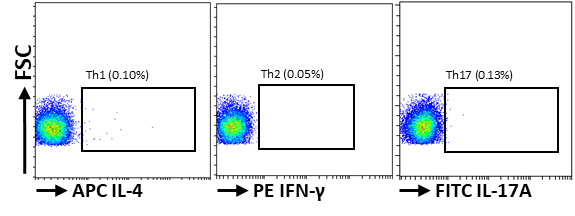


**Supplementary Fig.2 FMO for flow cytometry analysis of T helper cells.**

**References**

1. Brozek JL, Bousquet J, Baena-Cagnani CE, Bonini S, Canonica GW, Casale TB, et al. Allergic Rhinitis and its Impact on Asthma (ARIA) guidelines: 2010 revision. The Journal of allergy and clinical immunology. 2010;126(3):466-76.

2. Peng YQ, Qin ZL, Fang SB, Xu ZB, Zhang HY, Chen D, et al. Effects of myeloid and plasmacytoid dendritic cells on ILC2s in patients with allergic rhinitis. J Allergy Clin Immunol. 2019.

3. Thery C, Amigorena S, Raposo G, Clayton A. Isolation and characterization of exosomes from cell culture supernatants and biological fluids. Curr Protoc Cell Biol. 2006;Chapter 3:Unit 3 22.

4. Fang S-B, Zhang H-Y, Wang C, He B-X, Liu X-Q, Meng X-C, et al. Small extracellular vesicles derived from human mesenchymal stromal cells prevent group 2 innate lymphoid cell-dominant allergic airway inflammation through delivery of miR-146a-5p. Journal of Extracellular Vesicles. 2020;9(1):1723260.

5. Zhang F, Li R, Yang Y, Shi C, Shen Y, Lu C, et al. Specific Decrease in B-Cell-Derived Extracellular Vesicles Enhances Post-Chemotherapeutic CD8(+) T Cell Responses. Immunity. 2019;50(3):738-50.e7.

6. Fang SB, Zhang HY, Jiang AY, Fan XL, Lin YD, Li CL, et al. Human iPSC-MSCs prevent steroid-resistant neutrophilic airway inflammation via modulating Th17 phenotypes. Stem Cell Res Ther. 2018;9(1):147.
